# Prevalence and determinants of cervical cancer screening among women aged 30–49 years in Nigeria: A population-based cross-sectional study

**DOI:** 10.1371/journal.pgph.0006668

**Published:** 2026-06-16

**Authors:** Tope Olubodun, Pankras Luoga, Jovinary Adam

**Affiliations:** 1 Department of Community Medicine and Primary Care, Federal Medical Centre Abeokuta, Abeokuta, Ogun State, Nigeria; 2 Department of Development Studies, School of Public Health and Social Sciences, Muhimbili University of Health and Allied Sciences, Dar es Salaam, United Republic of Tanzania; University of Balamand Faculty of Health Sciences, LEBANON

## Abstract

Cervical cancer poses a major global health challenge, particularly impacting women.Although largely preventable through regular screening, uptake remains suboptimal in Nigeria due to multiple access barriers. Guided by Andersen’s Behavioural Model of Health Services Use – which posits that health service utilisation is influenced by predisposing, enabling, and need factors – this study assessed the prevalence and determinants of cervical cancer screening among women aged 30–49 years in Nigeria. We analysed secondary data from the 2023–24 Nigeria Demographic and Health Survey, including a weighted sample of 17,649 women aged 30–49 years. Analyses accounted for the complex survey design and were conducted using Stata version 18. Multivariable logistic regression was used to identify determinants of cervical cancer screening, with statistical significance set at *p* < 0.05. Cervical cancer screening among women aged 30–49 years in Nigeria was 5% (95% CI = 4.6, 5.5), ranging from 3.3% in the North East and 10.8% in the South West. Consistent with Andersen’s model, predisposing factors such as older age and higher education (AOR = 2.37; 95% CI: 1.58–3.57) were associated with increased screening. Enabling factors – including higher wealth quintile (AOR = 1.47; 95% CI: 1.03–2.14), health insurance coverage, recent health facility visit, prior breast cancer examination (AOR = 4.22; 95% CI: 2.70–5.91) and regular mass media exposure – significantly improved uptake. The Need factors – HIV-positive status (AOR = 4.34; 95% CI: 2.34–8.05) was a strong predictor. Conversely, higher parity (≥3 children) and residence in certain regions (North Central and South East) were associated with lower odds of screening. Cervical cancer screening prevalence among Nigerian women remains very low. Findings, highlight the critical role of socioeconomic access, health system contact, and perceived need in shaping screening behaviour. Interventions should prioritise reducing financial and geographical barriers, strengthening routine health system touchpoints, and enhancing risk perception through targeted health education to improve equitable screening uptake.

## Introduction

Cervical cancer is the fourth most common cancer among women globally [1]. Cervical cancer arises from infection with the human papillomavirus (HPV) and gradually progresses through identifiable pre-malignant stages, eventually leading to invasive cancer after several years [2]. The burden of cervical cancer is greatest in low- and middle-income countries, and cervical cancer is the commonest cancer among women in the African region [3]. In 2020, the UN General Assembly endorsed the WHO Global Strategy for the elimination of cervical cancer, which established the 90-70-90 targets to be achieved by 2030 [4]. Ninety per cent of adolescent girls should be vaccinated with the HPV vaccine by the age of 15 years, 70% of women should be screened for cervical cancer with a high-performance test at the age of 35years, and again at the age of 45 years, and lastly, 90% of women with pre-invasive disease should be treated and malignant disease managed [4]. The Global target stated that all countries should attain and maintain an ASIR of 4 per 100,000 cases of cervical cancer by 2030. However, in low- and middle-income countries, the age-standardised incidence rate (ASIR) of cervical cancer is 26.7 per 100,000 women, compared with 9.6 per 100,000 women in high- income countries [5]

In Nigeria, the largest country in Africa, the ASIR of cervical cancer is 18.4 per 100,000 [4]. About 12,000 women are diagnosed with cervical cancer each year, and there are 8,000 deaths from the disease each year in Nigeria [6]. These estimates likely underrepresent the true burden, given the country’s weak cancer surveillance and incomplete reporting systems. In Nigeria, women with cervical cancer frequently present at late stages, with attendant poor outcomes. A study at Lagos University Teaching Hospital reported that 70% of cervical cancer patients presented at FIGO Stage 3 and 4 [7]. A retrospective review of cases of cancer of the cervix at the Gynaecology Oncology Unit, Aminu Kano Teaching Hospital, Kano, in northern Nigeria, reported that 86.8% of patients presented in late stages [7]. The 5-year survival rate of women with cervical cancer was reported to be 31.7% in a study in Zaria, northwest Nigeria, and a retrospective cohort study in northcentral Nigeria reported an overall death rate of 79.8 per 100 women-years after a cumulative follow up of 526.17 months.

The Federal Ministry of Health in Nigeria, alongside partners like GAVI and WHO, launched routine HPV vaccination of adolescent girls in 2023, and to date, about 15 million girls have been vaccinated [8]. Vaccination, however, does not preclude the need for regular screening of eligible women, and again, most women in Nigeria did not receive the HPV vaccine as adolescents. Cervical cancer screening in Nigeria currently is fragmented. There are no organised cervical cancer screening programmes as is obtained in developed countries like the UK, Canada and Australia. There are infrequent cervical cancer screening outreaches in communities and health facilities organised by non-Governmental organisations, philanthropists, and state governments [9,10]. Facilities for cervical screening are generally sub-optimal in Nigerian health facilities, particularly the primary and secondary level health facilities, and tertiary health facilities serve as referral centres for cervical cancer screening and treatment [11].

The importance of cervical cancer screening cannot be overemphasised, as cervical cancer is largely preventable, and no woman should die from a preventable cause. Uptake of screening in Nigeria is abysmally low in Nigeria, with most studies reporting prevalence of screening less than 10% [12–17]. The Federal Ministry of Health is galvanising plans to strengthen cervical cancer screening in Nigeria. The National Strategic Plan for the Prevention and Control of Cancer of the Cervix in Nigeria 2023–2027 has a mandate to screen 50% eligible women aged 25–49 years using a high-performance test at least twice in their lifetime by 2027, with the age of eligibility starting at 30 years for the general population and 25 years for women living with HIV (WLHIV) [18]. Screening is to be integrated into other sexual and reproductive health services including family planning and HIV/AIDs programmes, and as a minimum, screening is recommended for every woman 25–65 years of age at least once in her lifetime [18].

Several studies have been carried out in Nigeria to assess the prevalence of cervical cancer screening and determinants, as well as women’s knowledge and attitudes towards screening [19–22]. Till date, there has been no study that presents nation-wide estimates of cervical cancer screening prevalence and determinants. We thus use data from the recent 2024 Nigeria Demographic and Health Survey (NDHS) to assess the prevalence of cervical cancer screening and the determinants of screening among Nigerian women. Unlike many sub-national studies, our study is limited to women aged 30–49years, who form the group of women eligible for screening, unlike previous studies that mostly include all women of reproductive age [19,22,23]. Thus, our study aims to provide precise and actionable estimates of cervical cancer screening in Nigeria. Understanding the prevalence and determinants of cervical cancer screening in Nigeria will serve as an important tool for advocacy and for planning interventions towards eliminating cervical cancer in Nigeria. This study aimed to assess the prevalence of cervical cancer screening and examine its determinants among women aged 30–49 years in Nigeria using Andersen’s Behavioural Model of Health Services Use.

### Theoretical framework for understanding the determinants of cervical cancer screening in Nigeria

This study was guided by Andersen’s Behavioural Model of Health Services Use, a widely applied framework for understanding determinants of healthcare utilisation. The model posits that health service use is influenced by three interrelated domains – predisposing factors, enabling factors, and need factors – which collectively shape health-seeking behaviour and subsequent health outcomes [24].

In this study, we employed an **adapted application of Andersen’s model**, tailored to the variables available in the Nigeria Demographic and Health Survey (NDHS) 2023–24 dataset. This adaptation preserves the core theoretical structure of the model while operationalising its constructs using proxy indicators measurable within the dataset.

Predisposing factors represent socio-demographic characteristics that influence an individual’s propensity to utilise health services. In this study, these included age, marital status, and educational level, which may shape awareness, knowledge, and attitudes towards cervical cancer screening.

Enabling factors refer to the resources and conditions that determine whether individuals are able to access healthcare services. In this study, these factors were considered across multiple levels. At the individual and household level, variables such as wealth status, employment status, and health insurance coverage reflect the financial capacity to seek care. At the community level, place of residence, geo-political zone, and distance to a health facility capture geographical and structural access to services. In addition, exposure to mass media represents access to health information, which can influence awareness and utilisation. Prior contact with the health system, such as visiting a health facility in the last 12 months, indicates opportunities for interaction with healthcare providers. Finally, service utilisation linkages—such as history of breast cancer examination and use of modern contraceptives—reflect points where cervical cancer screening can be integrated into existing health services. Together, these factors capture both the ability to access care and the opportunities to receive screening within routine health service use.

Need factors refer to the individual’s health status or underlying risk, which directly influences the decision to seek care. In this study, need factors included HIV status, parity, age at first sex, age at first birth, and smoking status. These variables serve as proxies for underlying health risk and potential perceived or evaluated need for cervical cancer screening, given the absence of direct measures of perceived health status in the NDHS.

**Fig 1:**
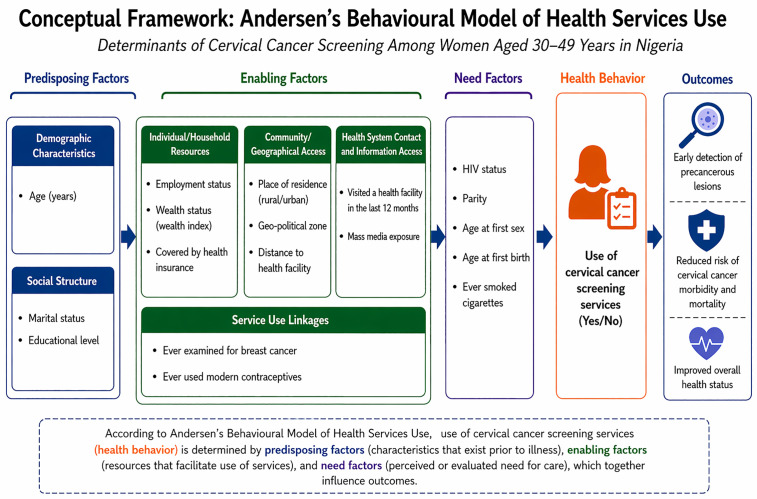
Conceptual framework: Andersen’s behavioural model of health services use.

Within this framework, the health behaviour of interest is the utilisation of cervical cancer screening services, while the expected outcomes include early detection of precancerous lesions, reduced cervical cancer morbidity and mortality, and improved overall health status. This adapted framework guided the selection, categorisation, and analysis of variables, and informed the interpretation of findings in line with established theory [24] (Fig 1).

## Methods

### Ethics statement

As this study involved secondary analysis of anonymised data, additional ethical approval was not required. Permission to access and download the datasets was obtained through registration on the MEASURE DHS website (https://dhsprogram.com/). For the primary survey (NDHS 2023–24), the study protocols were reviewed and approved by the ICF Institutional Review Board and the National Health Research Ethics Committee of Nigeria (NHREC). Informed consent was obtained from all participants, and all procedures were conducted in accordance with the Declaration of Helsinki.

### Study design and study population

We analysed secondary data from the 2023–24 Nigeria Demographic and Health Survey (2023–24 NDHS), which used a cross-sectional study design [25]. The study population were women of eligible age for cervical cancer screening in Nigeria [18]. Accordingly, analyses were restricted to a weighted sample of 17,649 women aged 30–49 years

### Data source and sampling strategy

Women recode data extracted from the Nigerian Demographic and Health Survey 2023–24 was analysed. The 2023–24 NDHS is the seventh Demographic and Health Survey conducted in Nigeria since 1990. Data collection was conducted from 1 December 2023–7 May 2024 [25]. The survey provides estimates of demographic and health indicators.

Nigeria is administratively divided into six geopolitical zones – the North Central, North East, North West, South East, South South, and South West. These zones comprise 36 states and the Federal Capital Territory (FCT), making a total of 37 subnational units. Each state is further divided into Local Government Areas (LGAs), which are subdivided into localities. For census demarcation, localities were partitioned into smaller, convenient units known as Enumeration Areas (EAs). In the 2023–24 NDHS, these EAs served as the basis for the primary sampling units (PSUs), also referred to as clusters [25].

The 2023–24 NDHS used a stratified two-stage sampling design. Each of the 36 states and the FCT was divided into urban and rural areas, creating 74 sampling strata. Samples were drawn independently within each stratum. In the first stage, clusters – defined as enumeration areas (EAs) – were selected with probability proportional to size within each stratum. A total of 1,400 clusters were selected, 701 in urban areas and 699 in rural areas. In the second stage, households were selected systematically. After listing all households in each chosen cluster, a fixed sample of 30 households per cluster was drawn with equal probability, yielding a total sample of about 42,000 households. All women age 15–49 who were either usual members of the selected households or visitors who stayed in the households the night before the survey were eligible to be interviewed [25].

### Study variables

#### Outcome variable.

During the survey, women of reproductive age were asked whether they had ever been screened for cervical cancer prior to the survey, which was measured using the question: “Ever tested for cervical cancer by a health care provider?”The binary variable was coded as “0” for “no” response and “1” for “yes” response, as was done by previous researchers using DHS data. There were no missing cases for the outcome variable.

#### Exposure variable.

The exposure variables were selected based on their availability in the NDHS dataset, evidence from prior studies [26,27], and their relevance within Andersen’s Behavioural Model of Health Services Use. Accordingly, variables were conceptually grouped into predisposing factors (age, marital status, educational level), enabling factors (employment status, wealth status, covered by health insurance, place of residence, geo-political zone, distance to health facility, visited a health facility in the last 12 months, ever examined for breast cancer, ever used modern contraceptive, mass media exposure,), and enabling factors (HIV status, age at first sex, age of respondent at first birth, parity, ever smoked cigarettes, as shown in Table 1. [26,27]

**Table 1:** Study variables description.

Variable	Description
**Predisposing factors**	
**Age (years)**	The age variable was divided into four categories in the NDHS dataset: 30–34, 35–39, 40–44, and 45–49. However, it was recoded into two categories: 30–39 and 40–49.
**Marital status**	Marital status was originally divided into six categories: never in union, married, living with partner, widowed, divorced, and no longer living together/separated. We recoded marital status into three categories for analysis: retaining the ‘never in union’ category, combining ‘married’ and ‘living with partner’ into the ‘married’ category, and combining ‘widowed,’ ‘divorced,’ and ‘no longer living together/separated’ into the ‘separated/widowed’ category
**Educational level**	Education level was utilized as recorded in the DHS dataset (No education, primary, secondary, higher)
**Enabling factors**	
**Employment status**	This variable was originally divided into eleven categories: not working, professional/technical/managerial, clerical, sales, agricultural (self-employed), agricultural (employee), household and domestic, services, skilled manual, unskilled manual, and other. For analysis, it was recoded into two categories: ‘unemployed’ for those not working, and ‘employed’ for all other categories
**Wealth status**	The wealth index variable was originally divided into five categories: poorest, poorer, middle, richer, and richest. However, it was recategorized into three groups for a more streamlined analysis: combining ‘poorest’ and ‘poorer’ into the ‘poor’ category, ‘richer’ and ‘richest’ into the ‘rich’ category, and retaining ‘middle’ as a separate third category
**Covered by health insurance**	This variable was used as recorded in the DHS dataset, with responses categorized as ‘Yes’ or ‘No.’
**Place of residence**	Place of residence was recorded in the DHS dataset as either urban or rural
**Geo-political zone**	This variable was utilized as recorded in the DHS dataset (North West, North East, North Central, South East, South South, South West)
**Distance to health facility**	This variable was utilized as recorded in the DHS dataset (not a big problem, big problem).
**Visited a health facility in the last 12 months**	This variable was utilized as recorded in the DHS dataset (yes, no).
**Ever examined for breast cancer**	This variable was recoded as ‘Yes’ for those who have ever been examined for breast cancer and ‘No’ for those who have never been examined
**Ever used modern contraceptives**	This variable was recoded into two categories: ‘No’ if a woman does not use any modern contraceptive methods, and ‘Yes’ if she does
**Mass media exposure**	The variable for mass media exposure combined radio, television, and newspapers. This was recoded into three categories: ‘Not at all,’ ‘Less than once a week,’ and ‘At least once a week.’
**Need factors**	
**HIV status**	This variable of HIV status was recoded as Negative and positive
**Age at first sex**	Age at first sex was recoded into three categories: ≤ 18, 19–29, and 30 and above.
**Age of respondent at 1st birth (years)**	This variable was originally a numerical variable, with values ranging from 10 to 47. We recoded into three categories: ≤ 19 years, 20–29 years, and 30 years and above.
**Parity**	This variable was originally a numerical variable, with values ranging from 0 to 16. We recoded into four groups: 0, 1–2, 3–4, and 5 and above.
**Ever smoked cigarettes**	This variable was utilized as recorded in the DHS dataset (yes, no)

### Statistical analysis

Data analysis was done with Stata version 18 (StataCorp LLC, College Station, TX, USA). Descriptive analysis was conducted using weighted frequencies and percentages to determine the prevalence of cervical cancer screening and to summarise participants’ background characteristics. The “*svyset*” command was used in STATA for assigning the sample weight and to adjust for the clustering effect and sample stratification. All data were weighted using the Individual (Women’s) Recode file weight (v005 divided by 1,000,000) in accordance with DHS guidelines to account for complex survey design and non-response [28]. The chi-square test was used to examine the association between the outcome variable (ever had cervical cancer screening) and independent variables. All variables with a p-value < 0.25 in the bivariate analyses were included in the multivariable logistic regression model. Using the variance inflation factor (VIF), multicollinearity between the independent variables was assessed. All VIF values were below the commonly accepted threshold of 10, with the highest being 3.6, indicating no significant multicollinearity among predictors. Multicollinearity test was performed among independent variables to avoid distortion of effect estimates due to high correlation among independent variables. Univariate analysis was also included between the exposure variables and the outcome variable – ever had cervical cancer screening.

All the independent variables were included in the multivariable logistic regression model as all *p*-values were less than 0.25. At the multivariate analysis, variables with p-values less than 0.05 were considered to be statistically significantly associated with the cervical cancer screening. The strength of the association was assessed using the adjusted odds ratio (AOR) along with its corresponding 95% confidence interval (CI).

## Results

### Background characteristics of the study respondents

A total sample of 17,649 women aged 30–49 years were involved in the analysis for this study. The majority of the study respondents (59.4%) were aged 30–39 years. About three-quarter of the women (76.2%) were employed. Half of the study respondents (50.1%) resided in rural areas. Nearly half of the study participants (46.3%) were from rich households. The characteristics of the respondents are presented in Table 2.

**Table 2:** Background characteristics of women aged 30–49 years in Nigeria (N = 17,649) (Weighted sample).

Variables	Frequency	Percentage
**Predisposing factors**		
**Age (years)**		
30-39	10,482	59.4
40-49	7,167	40.6
**Marital status**		
Never in union	685	3.9
Married	15,496	87.8
Separated/widowed	1,468	8.3
**Educational level**		
No education	6,788	38.5
Primary	2,377	13.5
Secondary	5,535	31.3
Higher	2,949	16.7
**Enabling factors**		
**Employment status**		
Unemployed	4,193	23.8
Employed	13,456	76.2
**Wealth status**		
Poor	6,175	35
Middle	3,293	18.7
Rich	8,181	46.3
**Covered by health insurance**		
No	16,839	95.4
Yes	810	4.6
**Place of residence**		
Rural	8,844	50.1
Urban	8,805	49.9
**Geo-political zone**		
North West	5,011	28.4
North East	2,652	15.0
North Central	3,106	17.6
South East	1,660	9.4
South South	2,242	12.7
South West	2,976	16.9
**Distance to health facility**		
Big problem	4,450	25.2
Not a big problem	13,199	74.8
**Visited a health facility in the last 12 months**		
No	10,258	58.1
Yes	7,391	41.9
**Ever examined for breast cancer**		
No	16,246	92
Yes	1403	8
**Ever used modern contraceptives**		
No	14,571	82.6
Yes	3,078	17.4
**Mass media exposure**		
Not at all	6,571	37.2
Less than once a week	3,350	19.0
At least once a week	7,728	43.8
**Need factors**		
**HIV status**		
Negative	7,420	98.9
Positive	87	1.1
**Age at first sex**		
≤18	11,880	67.3
19-29	5,661	32.1
30 and above	108	0.6
**Age of respondent at 1st birth**		
≤19	6,948	41.5
20-29	8,583	51.3
30 and above	1,203	7.2
**Parity**		
0	915	5.2
1-2	2,787	15.8
3-4	5,290	30
5 and above	8,657	49
**Ever smoked cigarettes**		
No	17,578	99.6
Yes	71	0.4

### Cervical cancer screening among women aged 30–49 years across zones in Nigeria

**Fig 2:**
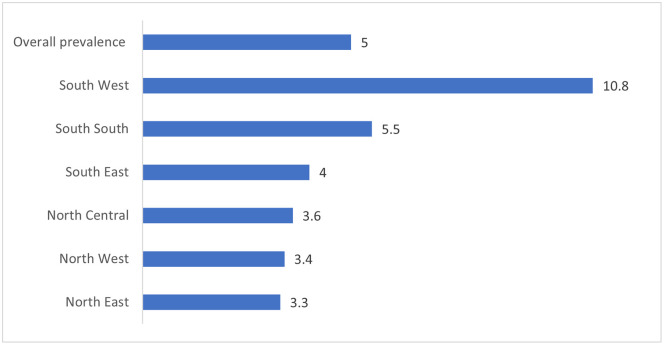
Prevalence of cervical cancer screening among women aged 30-49 years across zones in Nigeria (%).

The study found that overall cervical cancer screening among women aged 30–49 years in Nigeria was 5% (95% CI = 4.6, 5.5). Cervical cancer screening varied across zones, highest in the South West (10.8%), and lowest in the North East(3.3%) (Fig 2).

### Bivariate analysis of cervical cancer screening and independent variables

The bivariate analysis showed that the proportion of women screened for cervical cancer increased with age, from 4.4% [95% CI: 3.9, 5.0] among those aged 30–39 years to 5.8% [95% CI: 5.2, 6.5] among women aged 40–49 years The proportion of women that had been screened also increased with education level, from 2.2%[95% CI: 1.7, 2.8] among women with no education to 12.5% [95% CI: 11.1, 14] among those with higher education. The percentage of women who had received cervical cancer screening increased with household wealth, from 2.1% [95% CI: 1.7, 2.7] among those in the poor households to 8% [95% CI: 7.2, 8.8] among those in the rich households. Women residing in urban areas had higher proportion of cervical cancer screenings (7%, 95% CI: 6.3, 7.8) compared to those in rural areas (3%, 95% CI: 2.5, 3.5). Women who reported that distance to a health facility was not a major problem had higher screening uptake (5.6%, 95% CI: 5.1, 6.1) than those who perceived distance as a major barrier (3.3%, 95% CI: 2.7, 4.0). Women living with HIV had much higher screening prevalence (20.7%, 95% CI: 13.1, 30.9) than HIV-negative women (8.5%, 95% CI: 7.8, 9.4). (Table 3**).**

**Table 3:** Bivariate analysis of cervical cancer screening and independent variables among women aged 30–49 years in Nigeria.

Variables	Percentage of women screened	95% CI	p-value
**Predisposing factors**			
**Age (years)**			**<0.001**
30-39	4.4	[3.9, 5.0]	
40-49	5.8	[5.2, 6.5]	
**Marital status**			**<0.001**
Never in union	5.2	[3.7, 7.2]	
Married	4.8	[4.4, 5.3]	
Separated/widowed	6.6	[5.2, 8.2]	
**Educational level**			**<0.001**
No education	2.2	[1.7, 2.8]	
Primary	2.9	[2.2, 3.9]	
Secondary	5.3	[4.6, 6.1]	
Higher	12.5	[11.1, 14]	
**Enabling factors**			
**Employment status**			**<0.001**
Unemployed	3.1	[2.4, 3.8]	
Employed	5.6	[5.1, 6.1]	
**Wealth status**			**<0.001**
Poor	2.1	[1.7, 2.7]	
Middle	3	[2.3, 3.9]	
Rich	8	[7.2, 8.8]	
**Covered by health insurance**			**<0.001**
No	4.4	[3.9, 4.8]	
Yes	18	[15.1, 21.3]	
**Place of residence**			**<0.001**
Rural	3	[2.5, 3.5]	
Urban	7	[6.3, 7.8]	
**Geo-political zone**			**<0.001**
North West	3.4	[2.7, 4.3]	
North East	3.3	[2.4, 4.5]	
North Central	3.6	[2.9, 4.5]	
South East	4	[3.2, 4.9]	
South South	5.5	[4.5, 6.6]	
South West	10.8	[9.3, 12.5]	
**Distance to health facility**			**<0.001**
Big problem	3.3	[2.7, 4.0]	
Not a big problem	5.6	[5.1, 6.1]	
**Visited health facility last 12 months**			**<0.001**
No	3.6	[3.1, 4.1]	
Yes	7	[6.2, 7.8]	
**Ever examined for breast cancer**			**<0.001**
No	2.5	[2.2, 2.9]	
Yes	33.4	[30.4, 36.5]	
**Ever used modern contraceptive**			**<0.001**
No	4.6	[4.2, 5.1]	
Yes	6.9	[5.9, 8.2]	
**Mass media exposure**			**<0.001**
Not at all	2.1	[1.7, 2.6]	
Less than once a week	4.6	[3.8, 5.6]	
At least once a week	7.6	[6.9, 8.4]	
**Need factors**			
**HIV status**			**<0.001**
Negative	8.5	[7.8, 9.4]	
Positive	20.7	[13.1, 30.9]	
**Age at first sex**			**<0.001**
≤18	4	[3.5, 4.5]	
19-29	7	[6.3, 7.9]	
30 and above	8.2	[4.3, 15.1]	
**Age of respondent at 1st birth**			**<0.001**
≤19	3.3	[2.7, 3.9]	
20-29	5.3	[4.7, 5.9]	
30 and above	9.8	[8.1, 11.9]	
**Parity**			**<0.001**
0	8.8	[6.9, 11.2]	
1-2	8.7	[7.4, 10.1]	
3-4	5.8	[5.1, 6.6]	
5 and above	2.9	[2.5, 3.4]	
**Ever smoked cigarettes**			0.0533
No	5	[4.6, 5.4]	
Yes	10	[4.9, 19.4]	

### Determinants of cervical cancer screening among women aged 30–49 years in Nigeria

Women aged 40–49 years (AOR = 1.51; 95% CI = 1.24, 1.83) had higher odds of cervical cancer screening compared to those aged 30–39 years. Women with higher than secondary education (AOR = 2.37; 95% CI = 1.58, 3.57), had higher odds of cervical cancer screening compared to women with no education. The odds of cervical cancer screening were higher among women from the rich quintile (AOR = 1.47; 95% CI = 1.03, 2.14) compared to women from poor households. Women covered by health insurance (AOR = 2.04, 95% CI = 1.59, 2.61) had higher odds of cervical cancer screening compared with those without health insurance. Women who visited health a facility in the last 12 months (AOR = 1.65, 95% CI = 1.36, 1.99), women who were exposed to mass media at least once a week (AOR = 1.69, 95% CI = 1.18, 2.43), women who have ever been examined for breast cancer (AOR = 4.22, 95% CI = 2.7, 5.91), and women who were living with HIV (AOR = 4.34, 95% CI = 2.34, 8.05), were more likely to have ever undergone cervical cancer screening. Furthermore, women with 3–4 children (AOR = 0.75, 95% CI = 0.59, 0.95), and those with 5 or more children (AOR = 0.57, 95% CI = 0.42, 0.78), and women from the North Central (AOR = 0.60, 95% CI = 0.42. 0.84, and South East zones (AOR = 0.65, 95% CI = 0.45, 0.93) had lower odds of cervical screening in Nigeria as presented in Table 4.

**Table 4:** Multivariable logistic regression analysis of factors associated with cervical cancer screening among women aged 30–49 years in Nigeria.

Variable	COR	95% CI	p-value	AOR	95%CI	p-value
**Predisposing factors**						
**Age (years)**						
30-39	ref			ref		
40-49	1.32	1.13 - 1.55	<0.001	1.51	1.24 - 1.83	**<0.001**
**Marital status**						
Never in union	ref			ref		
Married	0.93	0.65 - 1.32	0.684	1.56	0.75 - 3.24	0.239
Separated/widowed	1.29	0.84 - 1.97	0.248	2.01	0.93 - 4.34	0.075
**Educational level**						
No education	ref			ref		
Primary	1.34	0.91 - 1.96	0.139	0.91	0.58 - 1.41	0.672
Secondary	2.50	1.88 - 3.31	**<0.001**	1.38	0.94 - 2.04	0.099
Higher	6.31	4.80 - 8.30	**<0.001**	2.37	1.58 - 3.57	**<0.001**
**Enabling factors**						
**Employment status**						
Unemployed	ref			ref		
Employed	1.87	1.46 - 2.39	**<0.001**	1.03	0.78 - 1.36	0.834
**Wealth status**						
Poor	ref			ref		
Middle	1.43	0.98 - 2.09	0.061	1.03	0.66 - 1.61	0.892
Rich	4.02	3.08 - 5.25	**<0.001**	1.47	1.03 - 2.14	**0.042**
**Covered by health insurance**						
No	ref			ref		
Yes	4.80	3.83 - 6.02	**<0.001**	2.04	1.59 - 2.61	**<0.001**
**Place of residence**	1.43	0.98 - 2.09	0.061			
Rural	Ref			ref		
Urban	2.43	1.99 - 2.98	**<0.001**	0.94	0.73 - 1.20	0.605
**Geo-political zone**						
North West	ref			ref		
North East	0.97	0.65 - 1.44	0.873	0.98	0.64 - 1.48	0.906
North Central	1.06	0.77 - 1.46	0.712	0.60	0.42 - 0.84	**0.003**
South East	1.17	0.85 - 1.61	0.333	0.65	0.45 - 0.93	**0.019**
South South	1.65	1.22 - 2.23	**<0.001**	0.78	0.55 - 1.10	0.160
South West	3.42	2.59 - 4.52	**<0.001**	1.33	0.96 - 1.83	0.082
**Distance to health facility**						
Big problem	ref			ref		
Not a big problem	1.74	1.38 - 2.18	**<0.001**	1.08	0.85 - 1.36	0.538
**Visited a health facility in the last 12 months**						
No	ref			ref		
Yes	4.80	3.83 - 6.02	**<0.001**	1.65	1.36 - 1.99	**<0.001**
**Ever examined for breast cancer**						
No	ref			ref		
Yes	5.22	3.94 - 7.17	**<0.001**	4.22	2.73 - 5.91	**<0.001**
**Ever used modern contraceptives**						
No	ref			ref		
Yes	1.55	1.27 - 1.90	**<0.001**	1.03	0.82 - 1.28	0.819
**Mass media exposure**						
Not at all	ref			ref		
Less than once a week	2.28	1.69 - 3.07	**<0.001**	1.33	0.90 - 1.97	0.155
At least once a week	3.86	3.03 - 4.92	**<0.001**	1.69	1.18 - 2.43	**0.004**
**Need factors**						
**HIV status**						
Negative	ref			ref		
Positive	2.79	1.61 - 4.86	**<0.001**	4.34	2.34 - 8.05	**<0.001**
**Age at first sex**						
≤18	ref			ref		
19-29	1.82	1.53 - 2.16	**<0.001**	0.96	0.76 - 1.21	0.720
30 and above	2.15	1.07 - 4.33	**0.032**	0.46	0.16 - 1.36	0.161
**Age of respondent at 1st birth**						
≤19	ref			ref		
20-29	1.66	1.34 - 2.06	**<0.001**	0.90	0.70 - 1.16	0.416
30 and above	3.23	2.45 - 4.27	**<0.001**	0.97	0.67 - 1.39	0.856
**Parity**						
0	ref			ref		
1-2	0.98	0.73 - 1.33	0.914	0.77	0.62 - 1.20	0.691
3-4	0.64	0.48 - 0.85	**0.002**	0.75	0.59 - 0.95	**0.016**
5 and above	0.31	0.23 - 0.43	**<0.001**	0.57	0.42 - 0.78	**<0.001**
**Ever smoked cigarettes**						
No	ref			ref		
Yes	2.12	0.97 - 4.62	0.059	2.18	0.93 - 5.12	0.073

COR – crude odds ratio, AOR – adjusted odds ratio.

## Discussion

This study used data from the 2023–24 NDHS to assess the prevalence and determinants of cervical cancer screening among women eligible for cervical cancer screening, aged 30–49 years in Nigeria, guided by Andersen’s Behavioural Model. The prevalence of cervical cancer screening was five per cent and ranged from three per cent in the northeastern part of the country to about eleven per cent in the southwest. Older women, women with tertiary education, women of the rich wealth status, and women who have health insurance were more likely to have been screened. Also, women living with HIV, and women who had been screened for breast cancer, and women who had visited a health facility in the last 12 months, had higher odds of cervical cancer screening. Conversely, marital status, employment status, place of residence, distance to health facility, use of contraceptives, smoking history and age at first birth were not found to be predictors of cervical cancer screening among Nigerian women.

The low prevalence of cervical cancer screening seen in our study has also been reported from sub-national studies across the country [22,23,29,30]. In a study conducted in urban areas of Ilorin, northcentral Nigeria, only 8% of women had been screened for cervical cancer [23]. In a facility-based study in Ibadan, southwest Nigeria, also, only 9% of women had been screened for cervical cancer [22]. Screening uptake has also been shown to be very low in rural areas and slum areas in Nigeria. Only four per cent of women in a rural community in south-south Nigeria [29], and less than one per cent in a slum community in Lagos [30] had ever been screened for cervical cancer.

In contrast to our findings, the prevalence of screening among immigrant women in Ontario, Canada, was 53.1% [31], and in Texas, 79% of women reported receiving Pap screening in the past 3 years [32]. The prevalence of cervical cancer screening reported in our study is, however, similar to some other countries in Africa. A nationwide study in Ghana reported a prevalence of seven per cent [33] and a country-wide study in Côte d’Ivoire, also reported a prevalence of seven per cent [34]. In East Africa, a secondary analysis of the Kenyan demographic health survey reported a prevalence of about seventeen per cent [35]and in Tanzania, a prevalence of seven percent was reported [36]. The low prevalence of cervical cancer screening observed in our study and seen across other African countries is not unassociated with the lack of organised cervical cancer screening programmes in these countries. In addition, in Nigeria, facility-level barriers such as lack of cervical cancer screening facilities across primary, secondary and tertiary health facilities, shortage of health workforce, sociocultural barriers, low level of screening awareness and fear of receiving bad results have been documented to contribute to low screening rates [11,37,38].

### Predisposing factors

In this study, women aged 40–49 years had slightly higher odds of screening compared with those aged 30–39 years. This pattern was also observed in a study that analysed national data from four African countries [39], and also in a nationwide study in Cambodia [40]. This finding may be due to a cumulative effect of increasing age as older women could have had more contact with the health system. Older women could also perceive themselves as more susceptible to cancer. Within Andersen’s model, age functions as a predisposing characteristic that can influence health beliefs and the propensity to seek care.

Higher odds of being screened were observed among women with tertiary education, and also among women belonging to the rich wealth tertile. Education, as a key social structure factor in Andersen’s model, enhances individuals’ capacity to recognise the benefits of screening and navigate the health system. In a study carried out in Ghana that applied Andersen’s model as well, education status was also found to be a predictor of cervical cancer screening [41] Health-seeking behaviour is generally better among women of higher educational status due to better health enlightenment. For example, women with higher educational status in Nigeria are more likely to attend antenatal care, deliver their babies in health facilities and completely immunise their children [42–45]. Some studies done to assess determinants of cervical cancer screening in different settings in Nigeria, however, have found no statistically significant association between level of education, and uptake of cervical cancer screening. Notably, these studies reported very low prevalence of screening and had relatively small sample sizes, hence may not have been adequately powered to detect such a difference [22,23,46].

### Enabling factors

Enabling resources played a significant role in determining screening uptake. Women in the higher wealth quintile were more likely to be screened in our study. This may be because they are more likely to afford screening costs. Similar findings have also been reported in other population-based studies assessing determinants of cervical cancer screening in low- and middle-income settings in Africa and Asia [33,36,47,48]. Also, a study conducted in the United States of America, among Hispanic and African-American women, guided by Andersen’s Behavioural Model of Health Services Use, identified affordability of screening services as a key determinant of cervical cancer screening uptake [49].

Having health insurance was a significant predictor of cervical cancer screening in our study, with insured women being twice as likely to be screened as uninsured women. In Haiti [50]and in Tanzania [36]as well, insured women were more likely to be screened than uninsured women. In Nigeria, the coverage of health insurance is very low at 3% [51]. This low coverage of health insurance was evident in our study as only 4.6% of women were covered by health insurance. Although the National Cancer Control Plan recommends expanding access to screening in Nigeria [18], at present, cervical cancer screening is not covered by the National Health Insurance Scheme and State Health Insurance Schemes, although some private health insurance schemes do cover cervical cancer screening. The association observed in our study regarding health insurance and cervical cancer screening may be a mirror of better health seeking behaviour and health awareness among women on health insurance.

Women who had visited a health facility in the last 12 months were more likely to be screened, suggesting that recent contact with the health system creates important opportunities for the uptake of preventive services such as cervical cancer screening. Women who interact with healthcare providers are more likely to receive health education, counselling, or recommendations for screening, either through routine consultations or opportunistic screening initiatives. Such encounters may also enhance awareness, reduce misconceptions, and strengthen the perceived need for screening. Similar finding to our study was reported by Agsedom et al. in Tanzania [36] and Adzigbli et al. in Ghana [33], where screening rates were higher among women with contact with the healthcare system in the last 12 months.

Women who had been screened for breast cancer in our study were also more likely to have been screened for cervical cancer. This may be partly because many cancer screening outreaches organised in Nigerian communities, by state governments and non-governmental organisations, focus on screening women for both breast cancer and cervical cancer [9,10]. Also, for women who have been screened as part of a general checkup or as a form of opportunistic screening, cervical cancer screening is often done alongside breast cancer screening [10]. Shared determinants of breast and cervical cancer screening, such as health literacy, access to care, and preventive health orientation, may also explain our findings. Similar finding was reported in Kenya [35] and in a meta-analysis from Ethiopia [52] where women screened for breast cancer were also more likely to have undergone cervical cancer screening.

Unlike the other health system contact enabling factors (visited a health facility in the last 12 months, and ever had a breast examination), contraceptive use was not found to be a predictor of cervical cancer screening. It is recommended that cervical cancer screening be integrated into reproductive health services [53,54]. This is, however, not obtained in most settings in Nigeria [11]. In Nigeria, “cues to action” that involve bringing health services closer to the users have been shown to be beneficial in increasing uptake rates of health services, including HIV screening in pregnancy [55] and HPV vaccination of adolescent girls [56]. As the prevalence of cervical cancer screening is very low in Nigeria, a similar approach can prove useful to increase cervical cancer screening rates. When women visit health facilities to assess contraception, this opportunity should also be utilised for health education and the provision of cervical cancer screening.

Exposure to mass media such as television, radio and newspapers was found to be a determinant of cervical cancer screening in this study. Women with regular exposure to mass media were more likely to have undergone screening, underscoring the role of information dissemination and health communication in shaping preventive health behaviours. Similar findings have been reported in other African countries [57,58]. Mass media platforms can increase awareness of cervical cancer, improve knowledge of available screening services, and help address misconceptions and fears surrounding screening procedures. Within Andersen’s Behavioural Model, mass media exposure represents an enabling factor by improving access to health information and facilitating informed decision-making. Mass media should be harnessed as a strategic component of cervical cancer prevention programmes, particularly in settings with low screening coverage.

Unlike what has been reported in some DHS studies in Africa [36,39], place of residence was not found to be a predictor of cervical cancer screening in our study. This shows that women residing in urban areas also have very low screening rates like those in rural areas in Nigeria, and this calls for increased sensitisation on cervical cancer screening as well improved access to screening services in both rural and urban settings. Within Andersen’s Behavioural Model, this suggests that geographic enabling factors alone are insufficient to drive utilisation without complementary financial and informational resources.

Similarly, distance to health facility was not found to be of cervical cancer screening in this study. This depicts that in Nigeria, even women who have better access to health facilities, also have low screening rates. Our finding contrasts with studies from other African settings where women for whom distance was not a big problem were more likely to be screened [57,58]. Our findings show the need for increased sensitisation and enlightenment of women on the importance of cervical cancer screening in Nigeria. This further highlights that enabling access must be reinforced by perceived need and awareness to translate into actual service use.

### Need factors

Our study reported that women living with HIV (WLHIV) were four times more likely to have received cervical cancer screening. The risk of cervical cancer is higher among HIV infected women due to immunosuppression. WLHIV often receive cervical cancer screening as part of their care, in many centres in Nigeria, in recent years. PEPFAR and APIN have played a key role in expanding cervical cancer screening for WLHIV in Nigeria by funding and integrating VIA-based screening and treatment into HIV clinics, training healthcare workers, and strengthening referral systems [59]. These efforts, as well as those of other non-governmental organizations have improved access to cervical cancer screening services among this group of women [59,60]. Within Andersen’s Behavioural Model, HIV status represents a strong evaluated need, while the integration of screening into HIV care enhances access, thereby significantly increasing utilisation.

In this study, established risk factors for cervical cancer, such as smoking, early age at first birth and high parity, did not translate to increased screening uptake. On the contrary, women who had more children were less likely to be screened. Our findings suggest that cervical cancer screening is low overall, and even women at higher risk are not being screened. From an Andersen’s model perspective, this indicates a disconnect between evaluated need (objective risk) and perceived need, which is critical in driving health-seeking behaviour. This also highlights the need for enhanced awareness about cervical cancer risk factors and the importance of ensuring that sexually active women correctly perceive their susceptibility and the value of screening. Addressing this gap will require targeted health education and risk communication strategies to transform underlying risk into perceived need and ultimately into service utilisation.

### Implications for policy and practice

The persistently low prevalence of cervical cancer screening in Nigeria underscores an urgent need for coordinated and sustained policy action to expand access and improve uptake. Interventions must address gaps across enabling, predisposing, and need factors to improve service utilisation. Establishing an organised, population-based screening programme with clear national guidelines, defined target populations, and functional referral pathways should be prioritised. Integrating cervical cancer screening—preferably through a screen-and-treat approach—into HIV clinics, maternal and reproductive health services, and primary healthcare facilities would help minimise missed opportunities and strengthen continuity of care.

Expanding financial protection for preventive services is equally critical. The strong socioeconomic gradient observed in this study highlights the need to include cervical cancer screening within the benefit packages of the National Health Insurance Authority (NHIA) and State Social Health Insurance Schemes. Incorporating screening into publicly funded insurance packages would reduce out-of-pocket expenditure and promote equitable access. Effective implementation of the proposed mandatory health insurance scheme could further enhance population coverage.

Screening uptake remained low even among women better access to health facilities and women at higher risk. Sustained community education and demand-generation strategies are therefore essential to improve awareness, risk perception, and health-seeking behaviour. Leveraging mass media platforms can play a pivotal role in disseminating accurate information on cervical cancer risk and the benefits of routine screening, thereby supporting behaviour change and increasing screening uptake.

### Strengths and limitations

A major strength of this study is the use of nationally representative NDHS 2023–24 data, which employed rigorous sampling procedures and provides robust, generalisable estimates for women aged 30–49 years. The large sample size increased statistical power to detect key associations. Limiting the analysis to women within the guideline-recommended screening age also ensured more accurate and policy-relevant prevalence estimates.

This study is however, limited by its cross-sectional design, which prevents causal inference. Self-reported measures may be affected by recall and social desirability bias, and misclassification is possible in settings with low awareness of screening. Additionally, important variables such as screening awareness, facility-level readiness, and health system constraints were not captured in the NDHS. Despite these limitations, the study provides valuable national evidence to guide cervical cancer prevention efforts in Nigeria.

## Conclusion

This study demonstrates that cervical cancer screening uptake in Nigeria remains critically low, with only about five per cent of women aged 30–49 years having ever been screened. Regional disparities were observed, with the highest prevalence in the South West and the lowest in the North East. Screening uptake was significantly associated with socioeconomic status, exposure to mass media, and selected health-related factors, including HIV-positive status and prior breast cancer screening. Nevertheless, screening coverage remained uniformly low across all population subgroups.

These findings highlight the urgent need to address financial, informational, and structural barriers to cervical cancer screening in Nigeria. Integrating screening services into existing reproductive, maternal, and HIV care platforms, alongside strengthened financing mechanisms and targeted awareness strategies, is essential to improve uptake. A comprehensive approach that simultaneously strengthens enabling resources, enhances risk perception, and addresses social determinants will be critical for improving screening uptake. Such efforts are critical for reducing the burden of cervical cancer and accelerating Nigeria’s progress towards national and global cervical cancer elimination targets, while ensuring equitable access for all eligible women.
